# Management of Combat Vascular Injuries Using Modern Imaging: Are We Getting Better?

**DOI:** 10.1155/2013/689473

**Published:** 2013-01-10

**Authors:** Samy S. Nitecki, Tony Karram, Amos Ofer, Ahuva Engel, Aaron Hoffman

**Affiliations:** ^1^Department of Vascular Surgery, Rambam Health Care Campus and Faculty of Medicine, Technion Israel Institute of Technology, P.O. Box 9602, 31096 Haifa, Israel; ^2^Interventional Radiology Unit, Rambam Health Care Campus and Faculty of Medicine, Technion Israel Institute of Technology, 31096 Haifa, Israel

## Abstract

*Background*. Vascular injuries often result in life threatening hemorrhage or limb loss. When they present with a single entry or exit wound, surgery is immediately indicated. With multiple injuries, however, imaging such as CTA is necessary for diagnosis and choice of treatment. *Methods*. For all combat-related vascular cases admitted to our medical center during the Lebanon wars in 1982 and 2006, we compiled and compared presenting signs and symptoms, means of diagnosis, treatments, and results. *Results*. 126 patients with vascular injuries were admitted (87 in 1982, 39 in 2006). 90% were male; mean age of 29 years (range 20–53). All injuries were accompanied by insult to soft tissue, bones, and viscera. 75% presented with injury to arteries in the extremities. 75% of these patients presented with limb ischemia, and 25% sustained massive blood loss. Treatments included venous interposition graft, end-to-end anastomosis, venous patch, endovascular technique (only in 2006), and ligation/observation. Complications included thrombosis and wound infections. Mortality and amputations occurred only in 1982, and this may be attributed to the use of imaging, advanced technique, and shorter average time from injury to hospital (7 hours). *Conclusions*. We recommend CTA as the first line modality for diagnosis of vascular injuries, as its liberal use allowed for early and appropriate treatment. Treatment outcomes improved with fast and effective resuscitation, liberal use of tourniquets and fasciotomies, and meticulous treatment by a multidisciplinary team.

## 1. Patients and Methods

**Figure 1:**
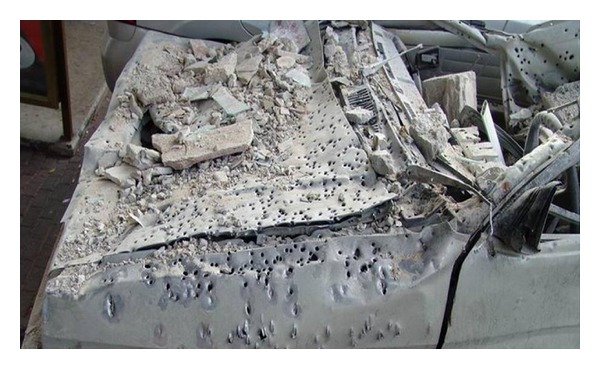
A car after fire of shrapnel or multiple pellets stored in long range missiles demonstrating the density and destruction. Imagine what would be the result in a human body.

During the first conflict in Lebanon, from June 6to September 17 1982, there were 2,176 wounded patients and during the 33-day conflict in Lebanon, from July 12 to August 15 2006, 511 wounded patients (soldiers and civilians) were admitted to our Emergency Room, out of which 87 (4%) and 39 (7.6%), respectively, had sustained a major vascular injury. There was male predominance of 92% wounded males and only 8% females. Their mean age was 29 years (range 20–53 years). All injuries were penetrating secondary to small arms used in direct combat, high velocity missiles, shrapnel, or multiple pellets stored in long range missiles (Figure 1). All injuries were accompanied by additional insult to soft tissue, bones, and viscera. Each patient suffering a vascular trauma had an average of 2.5 additional injuries. The mean injury severity score was 16 (range 9–38), similar in both groups.

Upon arrival to the ER of our level I trauma center, patients were examined, primarily treated, and underwent the obligatory scans (cervical spine and chest X-ray, FAST US scan). Next, they were sent either directly to the operation room or to radiology suits for additional studies such as CTA, angiography, or extremity X-rays, according to their condition assessment and diagnosis by the triage officer and the ER team.

Damage control resuscitation with permissive hypotension, minimal crystalloid, and early fast warmed blood transfusion were employed. 

Multidisciplinary approach was used to minimize ischemic time, set treatment priorities, determine the need for a temporary shunt, and allow for quick and meticulous vascular repair. Tourniquets were liberally used and removed in the operation room. Prior to surgery antibiotic treatment was started (penicillin 5 million and gentamycin 80 mg) and carried on for three days. Intraoperative systemic heparin (5,000 units) was routinely administered after a proximal control was achieved, prior to arterial cross clamping, except in cases of excessive bleeding due to other, non-vascular injuries. Vascular repairs were not left exposed. They were covered by a live and intact muscle flap or routed extra-anatomically outside a massive destructed soft tissue region. Following operation, Clexane was subcutaneously administered daily (40–80 mg) to prevent thromboembolic event in a non-mobile trauma patient. This was discontinued as the patients became ambulant. If contraindicated, because of bleeding, a temporary inferior vena cava (IVC) filter was introduced.

Postoperatively patients were treated in the intensive care unit to allow for convenient repeated operations, debridements of devitalized tissues. Wounds and distal pulses were checked on a daily basis. Ankle brachial indexes (ABI) were measured for nonpalpable or diminished extremity pulses by a handheld Doppler. A decrease of ABI in 0.2 resulted in a new vascular imaging by either CTA or angiography.

## 2. Results

Evacuation was faster during the 2006 war and mostly by helicopters. The average evacuation time were 16 hours in 1982, with 23% arriving 20 hours following the injury, and merely 7 hours in 2006 with only 4% arriving after 20 hours.

Hypovolemic shock was the presenting sign in 40–45% of the patients when first admitted in the emergency room.

Following resuscitation and primary assessment approximately 38% were rushed to the operation room, where diagnosis and treatment were done during surgery without utilizing any further imaging modality.

**Figure 2:**
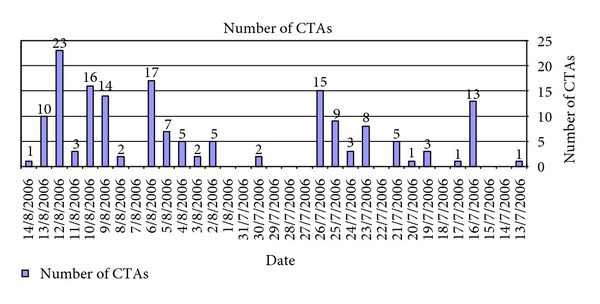
The actual number of patients arriving to perform vascular imaging on a daily basis. Too much for angiography to handle.

In 1982 angiography, a more time-consuming modality, was utilized for diagnosis. In sharp contrast, during the 2006 war CTA was the main diagnostic tool and angiography was utilized for endovascular treatment. Alltogether, CTA was performed in 166 patients (Figure 2) and was positive for a major vascular injury in 24 patients (14.5%). In one additional case, CTA was nonconclusive and the patient underwent angiography demonstrating an intact arterial tree.

Tourniquets are the basic equipment of every medical team to control battlefield hemorrhage. Nevertheless, unnecessary abuse may result in limb ischemia and paralysis, leading to amputation, and improper use does not stop the bleeding. In the Israeli Defense Forces, every soldier has a tourniquet that can be used when deemed necessary, even without the presence of a medical team. In our 2006 Lebanon War experience, tourniquets were liberally used in 39% of all extremity vascular injuries and were removed only in the operating room. Tourniquets were beneficial in 11 patients and overused (abused) in two patients without adverse effects.

The various injured vessels by their anatomic location are presented in Table 1. Arteries of the extremities (upper and lower) were, by far, the most frequently encountered injuries in 72% and 78% in 2006 and 1982, respectively. Lower extremity arteries were injured in almost half of the cases (46–48%). Almost 80% of the patients, with extremity vascular injury, had skeletal fractures. Limb ischemia was present in three quarters of extremity arterial injuries and massive bleeding in a quarter of the injuries.

Injured arteries were repaired by the methods summarized in Table 2. Endovascular technique was exclusively employed in 2006 for the treatment of five cases (13%): two cases of traumatic arteriovenous fistula of the internal iliac vessels, insertion of a stent-graft to the subclavian artery and two embolizations (maxillary artery and superior gluteal artery). A lateral repair or a patch was done in 15%–18%, end to end anastomosis was employed in quarter of the cases, and a venous interposition graft was performed in the majority of the cases (38–45%). Ligation only was done in 7–9% of the injuries of a single arm artery (radial or ulnar). Prior to ligation of one arm artery (radial or ulnar) the other artery was checked to be intact by a palpated pulse, Allen test and a normal flow by Doppler. No synthetic grafts or patches were used.

Open fasciotomy was liberally done for all cases of combined arterial and venous injury or for ischemia of a longer than 6 hours. Lower limb fasciotomy was performed in over three quarters of the patients with lower extremity injury (78–81%) followed later by a skin graft closure.

There were no mortalities and no amputations in 2006. In sharp contrast, there were three mortalities and two amputations in 1982 (Table 3). Their immediate postoperative course was relatively uneventful. Approximately two thirds of the patients had a second or even up to four operations for debridement of devitalized tissues. There were several cases of thrombosis (5–9%) mostly treated immediately by a new venous interposition graft. 

At followup, 5 years postoperatively, one above knee amputation was performed, in a patient with a well functioning interposition graft for popliteal artery and vein injury. The patient had paresis of his left leg due to severe sciatic nerve injury. He underwent arthrodesis of the knee joint but pus secreting wound and osteomyelitis necessitated amputation of the limb. All end to end anastomoses and patch vascular repairs were patent and had a normal flow pattern as assessed by duplex scan. In five interposition graft, a stenosis was encountered at the level of the distal anastomosis and treated by either open surgery or endovascularly. 

## 3. Discussion

The reported incidence of combat vascular trauma has been low in the past, with a range of only 0.2–4% [1–4]. This was probably due to excessive mortality during prolonged evacuation time. In more recent reports from the wars in Afghanistan and Iraq, this incidence has increased to 6.8% [5, 6], probably due to better immediate resuscitation and evacuation from the battleground. In our recent experience in the 2006 Lebanon War, the rate of vascular injuries increased to 7.6% of all casualties and to 10.8% in the subgroup of soldiers only [7]. 

Ballistic parameters are essential in the resulting tissue destruction. Internal and external ballistic are set by weapon muzzle velocity and bullet type. Terminal ballistics (wound ballistics), however, is the one seen by medical teams and the most important factor in the resulting injury. Cavity and bullet/pellet fragmentation results in further tissue destruction and blood vessels stretching and bursting [8–10]. 

Severe vascular injury is manifested by hemorrhage or ischemia and may harbor mortality or limb loss. Bleeding may be external or internal and lead to a hypovolemic shock which, indeed, was the presenting symptom in almost half of the cases in the present series. Therefore the surgeon's primary objective, in this setup, is to prevent exsanguination and death. Next to reestablish vascular supply (revascularization) to prevent amputation. 

Better understanding of physiologic derangement in combat sustained trauma resulted in further advancements in resuscitation such as permissive hypotension, minimal crystalloids infusion and early and rapid administration of warmed blood and its products to prevent physiologic deterioration (hypothermia and coagulopathy). Recent modern technologic improvements such as damage control, liberal use of tourniquets, fasciotomies, and temporary shunts enabled better life and limb salvage rates [11–16]. 

Vascular injuries with a single entry and/or exit wound, lead to surgery, immediately after the primary assessment and resuscitation in the emergency room. With multiple injuries, or the presence of only soft signs for a possible vascular injury, imaging is necessary before surgery, for both diagnosis and the choice of the preferred treatment.

Angiography is time consuming and sometimes really unfeasible, especially with over flow of multiple patients like in combat injuries.

CT scan, today, is routinely performed as part of the trauma patient work up. Intravenous administration of contrast medium, according to study protocol at the same time, provides enhancement of the arterial tree. 

**Figure 3:**
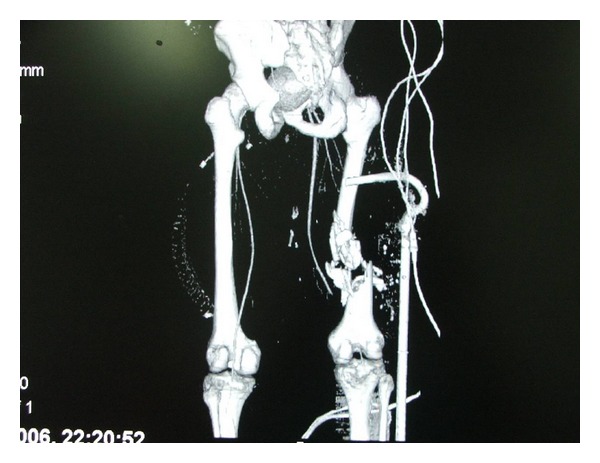
Three dimensional reconstruction of CTA demonstrating a left superficial femoral artery (injury) with a comminuted fracture. Note that after utilizing master cut and vessel extraction the splint does not interfere.

**Figure 4:**
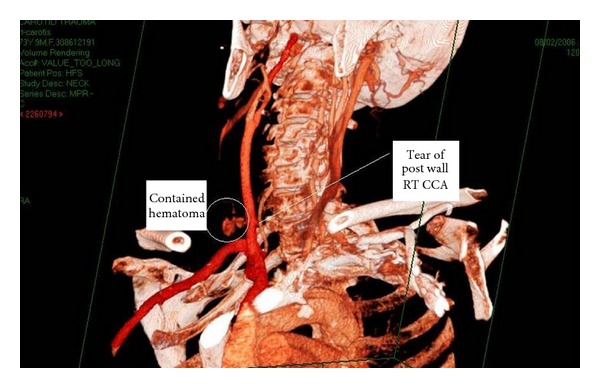
CTA demonstrating injury of the carotid artery with a tear and contained hematoma in the surrounding tissue.

Thus, CTA by virtue of its availability, quick data acquisition, and reconstruction becomes a major triage tool and angiography is utilized for endovascular treatment. CTA was demonstrated to be an accurate and reliable imaging modality in trauma of the carotids in our hands [17] and others [6]. CTA has a good resolution compared to angiography even for vascular injuries of the extremities [18, 19]. Conceivably CTA may not be diagnostic in the presence of multiple fragmentation casualties of the extremities due to metal artifacts. We were able to avoid these problems and demonstrate the reconstructed arterial tree by various methods such as volume rendering and maximal intensity projection (MIP) or vessel extraction and master cut (Figures 3 and 4). In the present series CTA was performed in 166 patients (Figure 2) out of which 24 patients (14.5%) were accurately diagnosed with a major vascular injury. In 141 patients CTA was able to exclude a vascular injury. In one additional case, CTA was non conclusive and the patient underwent angiography demonstrating an intact arterial tree. This was the only case in which angiography was used for diagnosis in the present study.

 Angiography, that is considered as the gold standard for diagnosing vascular injuries [10, 11], is problematic with multiple levels of extremity and fragmentation injuries. During wartime the number of simultaneously admitted patients in need of a vascular assessment is above and beyond the handling capability of invasive radiology teams and available angiography suits. Angiography is not feasible with mass casualties also because it is time consuming. During wartime the number, arrival times and severity of injuries of future patients are unknown and therefore quick assessment should be performed to allow readiness for the next wave of patients. During the Lebanon war there were six days with waves of 10 to 23 wounded patients waiting for imaging modality (Figure 2). Finally, angiography is demonstrating only the vessel lumen (actually lumenography) while CTA depicts not only the lumen but also the vessel wall and surrounding tissues. A massive injury of the surrounding tissues may play a roll in the strategy of vascular repair (i.e., the need for an extra-anatomic bypass).

A missed vascular injury (when the damage is not severe or overt) will present itself later as an arteriovenous fistula or a pseudoaneurysm [20]. 

Due to body armor extremities are more vulnerable and therefore vascular injuries of the extremities comprise approximately 60–90% of all injuries [1–12] and 72–78% in the present study. Exsanguination from extremity wounds is still the leading cause of preventable death in combat sustained injuries. The reported perioperative mortality in vascular casualties during the war in Afghanistan and Iraq was 4.3% [6]. There were no perioperative mortalities in the 2006 series although severe injuries and hypovolemic shock as the presenting sign occurred in almost half the patients. In sharp contrast there were three mortalities in the 1982 series. Our patients had similar anatomic distribution of vascular injuries (Table 1) compared to previously reported series.

During World War II, when poor physiologic and equipment conditions prevented arterial reconstruction, ligation was the accepted treatment [1] resulting in approximately 60% amputation rate. This amputation rate was dramatically reduced with arterial reconstruction to 13% during Korean War [2] and 12% in Vietnam [3]. The above mentioned advancements in assessment and treatment were employed in recent wars. Thus, in recent reports from Afghanistan and Iraq the amputation rates were improved to 4.2–9% [5, 6, 15]. 

In the present series with quick and meticulous treatment, contemplated priorities and multidisciplinary approach temporary shunts were employed in 17% and fasciotomies were performed in 78–81% of the patients. This resulted in 0% mortality and 0% amputation rate in the early postoperative period (only one amputation after 23 months). In the Lebanon war of 1982, with 87 patients over three months, we had 3% mortality and 2% amputation rate. At that time patients suffered from long evacuation period and less effective resuscitation compared to a front line hospital in Balad during the war in Iraq. The second Lebanon war, unlike the first one, twenty four years ago, was mostly an urban combat which interferes with radio control, injured identification, treatment, and evacuation.

Large extremity vein injuries usually accompany those of the adjacent arteries. Although their repair, especially when a complex venous reconstruction is need, is controversial because mostly they do not harbor life or limb loss, we believe that large injured veins should be repaired. Reconstructions of all large named veins were performed in the present series and have been reported on, separately [21]. 

The use of temporary shunts is still controversial in the literature. Eger et al. were among the first authors to report on the use of temporary vascular shunts in the modern era [22]. Although ischemia to an extremity has a lower treatment priority than massive hemorrhage, a temporary shunt may be inserted very quickly. The shunt serves for temporary revascularization by restoring blood flow to the leg, thereby reducing the ischemic time until the definitive vascular procedure is performed. Early shunting protects the extremity from further ischemic insult and reduces circulating markers of tissue injury.

Temporary shunt utilization for a major extremity vascular injury is recommended when other injuries assume first treatment priority or when prolonged evacuation/transportation times to a trauma center are expected. When dealing with a combined vascular, skeletal and soft tissue trauma, treatment priorities are dictated by limb ischemia. These injuries, especially when involving the popliteal artery, are associated with high limb loss rates. With a stable fracture, the vascular surgeon can perform the definitive repair followed by external or rigid internal fixation of the fracture. If, however, the fracture is unstable or the orthopedic surgeon needs to shorten the limb, a temporary vascular shunt is needed and the definitive vascular repair will be performed after the orthopedic treatment. Temporary vascular shunts in wartime casualties were used with beneficial effects by us and by others [22–24].

*Technique*. Injuries are exposed, proximal and distal control are achieved, vessel edges are debrided, thrombectomy is performed, heparinized saline is administered, and then a temporary shunt is inserted and secured in place. Shunts may be made of improvised plastic lines of different diameters or be specialized shunts, like the Javid Shunt for carotid repair. Possible problems include dislodgment and especially thrombosis in the range of 2–22% in both military and civilian patients and especially for shunts used in distal or very small arteries.

After control of bleeding, the vessel is inspected and the best method of repair is decided upon. Simple repairs of injured blood vessels are preferred. A segment of the injured vessel is resected if required. Lateral suture of lacerations and debridement of short segments with end-to-end anastomosis when possible are rapid and effective with few complications. Reversed venous bypasses are used when required, usually if the gap is more than 3 cm long. Multiple segments of injured vessels may be repaired by short venous segments. Synthetic grafts should be avoided if possible because of a higher risk of thrombosis and infection. Ligation of a major vessel should be used only in dying patients. All vascular repairs should be well covered by viable clean tissues to prevent late infection and bleeding.

In our recent experience, interposition reversed venous grafts were used in approximately 40% of cases, end-to-end anastomosis in a quarter, lateral repair or patch in 15–18%, while primary ligation was reserved for small arteries and for veins (7–9%). Extra-anatomic bypasses are rarely required.

The change in endovascular techniques enabled treatments that prior to that necessitated open surgery. 13% of the patients were treated by balloons, bare and covered stents or by embolization. 

Seemingly, casualties are complex with multiple fragmentations resulting in systemic effects and therefore today we are treating patients that would have never made it to care previously. The approach should be multidisciplinary in order to set treatment priorities and the needed repeated procedures for debridement of devitalized tissues. Vascular repairs should never be left uncovered, by a vital and healthy tissue even if a rotational flap is needed, because of infection and rupture. Intensive care monitoring for at least 24 hours is prudent. 

In conclusion, fast and effective resuscitation followed by rapid evacuation lends the injured a better chance at surviving. The hospital triage team should have a high index of suspicion for a vascular injury. A liberal use of a CTA should be practiced to allow for an early and accurate diagnosis of a vascular injury, even in the presence of multiple shrapnells and/or fragmentations. CTA, not angiography, should be the first line modality for the diagnosis of vascular injuries especially when dealing with mass casualties. Angiography should be reserved for endovascular treatment, not for diagnosis alone.

An aggressive, fast, meticulous, and repeated, if deemed necessary, treatment by a multi-disciplinary team leads to a greater life and limb salvage.

## Figures and Tables

**Table 1:** Arterial injuries by anatomic location.

Artery	Number (%)	
	2006 (%)	1982 (%)
Carotid	4 (10.25%)	3 (3.5%)
Innominate	1 (2.5%)	3 (3.5%)
Vertebral	1 (2.5%)	3 (3.5%)
Iliac	4 (10.25%)	8 (9.25%)
Gluteal	1 (2.5%)	2 (2.25%)
Subclavian	1 (2.5%)	4 (4.5%)
Brachial	4 (10.25%)	10 (11.5%)
Radial/Ulnar	5 (13%)	12 (14%)
Femoral	8 (20.5%)	18 (20.5%)
Popliteal	4 (10.25%)	10 (11.5%)
Tibial	6 (15.5%)	14 (16%)

Total	39 (100%)	87 (100%)

**Table 2:** Methods of repair.

	Number (%)	
	2006	1982
Vein interposition graft	39 (45%)	15 (38.5%)
End to end anastomosis	22 (25%)	9 (23%)
Lateral suture/patch	16 (18.5%)	6 (15.5%)
Ligation	8 (9%)	3 (7.5%)
Observation	2 (2.5%)	1 (2.5%)
Endovascular technique	0 (0%)	5 (13%)

Total	87 (100%)	39 (100%)

**Table 3:** Morbidity and mortality.

	2006 (%)	1982 (%)
Mortality	0 (0%)	3 (3.5%)
Amputation	0 (0%)	2 (2%)
Thrombosis	2 (5%)	8 (9%)
Infection	9 (23%)	34 (39%)
